# Gallbladder removal induces hepatic transcriptional and metabolic shifts with cholesterol dysregulation as a key feature

**DOI:** 10.1038/s41598-026-46659-8

**Published:** 2026-04-06

**Authors:** Feng Liang, Qiwu Yang, Lanfeng Xie, Jun Ma, Wenkai Zhao, Zhiyong Xiao, Zhen Wang, Caoyuan Li, Yilin Liu, Wei Chang, Suzhen Zhou, Zhaoxia Wu, Donghong Yan

**Affiliations:** 1 Department of General Surgery, The Affiliated People´s Hospital of Xinxiang Medical University, No. 63, Yiheng Street, Weibin District, Xinxiang, 453099 Henan Province China; 2 Department of Infectious Disease, Tongren Hospital, Shanghai Jiao Tong University School of Medicine, Shanghai, 200336, China; 3 Hongqiao International Institute of Medicine, Tongren Hospital, Shanghai Jiao Tong University School of Medicine, Shanghai, 200336, China

**Keywords:** Cholecystectomy, Liver, Transcriptomics, Metabolomics, Biomarkers, Computational biology and bioinformatics, Diseases, Gastroenterology, Genetics

## Abstract

The gallbladder, traditionally viewed as a digestive accessory organ for bile storage and concentration, has recently been implicated in post-injury liver recovery. Yet, the underlying pathophysiological mechanisms remain unclear. Here, we employed a cholecystectomized mouse model to investigate the systemic impact of gallbladder removal on the hepatic transcriptome and metabolome. Transcriptomic profiling revealed temporal shifts in liver responses: protein synthesis and processing pathways were activated 7 days post-cholecystectomy, while immune response activation and cholesterol metabolism dysregulation predominated by day 30. Parallel untargeted metabolomics identified 2,272 metabolites, with 111 and 125 significantly differentially expressed metabolites (DEMs) at days 7 and 30, respectively. The DEMs were primarily lipids and lipid-like molecules, as well as organoheterocyclic compounds. Integrated multi-omics analysis highlighted cholesterol metabolism as the most markedly altered pathway in the liver following cholecystectomy. Together, these results demonstrate the broad transcriptional and metabolic reshaping of the liver after gallbladder removal and position cholesterol homeostasis as a key mechanistic candidate in cholecystectomy-associated hepatic pathophysiology. Our findings provide new molecular insights into post-cholecystectomy liver adaptation and offer a foundation for future therapeutic strategies targeting related metabolic disorders.

**Supplementary Information:**

The online version contains supplementary material available at 10.1038/s41598-026-46659-8.

## Introduction

The gallbladder, an accessory organ of the digestive tract, serves the function of storing and concentrating bile in the absence of food. In response to ingestion of food, the gallbladder contracts and subsequently releases bile into the small intestine. This enables bile acids to enter the intestinal lumen, thereby facilitating the efficient digestion of dietary lipids. In typical circumstances, the preponderance of bile acids (approximately 95%) is reabsorbed from the intestine into the portal vein, where they are subsequently taken up by hepatocytes and reexcret into the bile. The gallbladder is a relatively well-conserved anatomical structure among mammals, exhibiting a high degree of variability in its presence and function, which is primarily influenced by dietary patterns. In species that consume food continuously, such as pigeons, rats and deer, the gallbladder is absent. This finding indicates that, in these species, bile is transferred directly from the liver to the intestine, bypassing the gallbladder as an intermediary. In contrast, the possession of a gallbladder is a characteristic that is universally observed in carnivorous species, with the exception of whales^1^.

Gallbladder disease is prevalent. The aforementioned ailments encompass gallstones, cholecystitis and gallbladder cancer^2^. Cholecystectomy is one of the most prevalent surgical procedures on a global scale. It is widely acknowledged as a safe procedure, with no discernible impact on metabolic regulation. The association between cholecystectomy and increased risk of metabolic syndrome and related complications, including non-alcoholic fatty liver disease (NALFD) and cirrhosis has been reported^3–5^. Moreover, previous data demonstrated that the basal metabolic rate and liver triglyceride content increases after cholecystectomy in mice^6,7^. However, it is important to note that the unnecessary cholecystectomy has been performed with considerable frequency on a global scale in order to facilitate the treatment of gallbladder diseases. This is due to the historical perception of the gallbladder’s role being confined to the concentration and storage of bile. It has been demonstrated in a number of studies that cholecystectomy has a carcinogenic effect on colon cancer and accelerates its progression^8^ and the latest research also unveils the unappreciated function of the gallbladder in the recovery of injured liver^9^. Consequently, it would be advantageous to conduct a more comprehensive investigation into the function and potential mechanisms of the gallbladder.

In this study, RNA-seq and metabolomics-based integrated analysis was employed to examine the potential pathophysiological mechanisms of the liver at early or late stages following cholecystectomy. Transcriptomics revealed that protein processing in the endoplasmic reticulum pathway was significantly activated in the early stages following cholecystectomy, due to the stress response triggered by organ removal, and mitochondrial energy metabolism and cholesterol catabolism are significantly suppressed by the late stage of cholecystectomy. Metabolomics showed that there was a disturbance in the homeostasis of lipids and organoheterocyclic metabolites in the Cocy7 group and the homeostasis of lipid metabolism, with a particular emphasis on cholesterol metabolism, was compromised in the Cocy30 group. The metabolomics study revealed a disruption in the homeostasis of lipids and organoheterocyclic metabolites in the Cocy7 group. In addition, the homeostasis of lipid metabolism, with a particular focus on cholesterol metabolism, was found to be impaired in the Cocy30 group. The integrated analysis revealed substantial metabolic alterations in cholesterol metabolism, steroid hormone biosynthesis, cortisol synthesis and secretion, ABC transporters, and bile secretion in cystectomised liver tissues. These findings not only expand our understanding of the effects of cholecystectomy on the liver, but also provide new clues for further research into the relationship between cholecystectomy and the risk of developing NALFD.

## Materials and methods

### Mice

Adult male 8-weeks-old C57BL/6 mice were obtained from Shanghai Lingchang Biotechnology Co., Ltd and acclimated for 7 days. The mice were maintained within a temperature-controlled chamber (5 per cage), the temperature of which was maintained at 21–25 °C, and the humidity at 45–65%. The mice were subjected to a 12-hour light/dark cycle, and had unrestricted access to food and water. Following a one-week acclimatization period, mice were randomly assigned to experimental groups using a computer-generated random number sequence (10 mice per time point for each group): the sham group, the Cocy7 group and the Cocy30 group. All mice were fed a standard laboratory chow (the diet composition was: 67.4% carbohydrate, 20.6% protein, and 12% fat). Water was available freely throughout the study period. All animal care was in accordance with the Guide for the Care and Use of Laboratory Animals, and experimental protocols were approved by the animal ethics committee of Tongren Hospital, Shanghai Jiao Tong University School of Medicine (No. A2025-016-01) and complied with the ARRIVE guidelines.

### Cholecystectomy

The cholecystectomy and sham operations were performed as previously described^7^. The mice were fasted for 10–12 h and underwent surgery under isoflurane anaesthesia (Isoflurane 1.5% inhalation anesthesia). Then, the abdominal skin was cleansed with 70% alcohol, and the abdominal cavity was accessed via a minor medial laparotomy. Using a cotton swab soaked in 0.9% sodium chloride solution, lift the liver so that its ventral surface adheres closely to the diaphragm, clearly exposing the hepatic hilum. Retract the intestines caudally to expose the bile duct. Apply two surgical ligatures with 4 − 0 silk thread around the cystic duct, then carefully resect the gallbladder. Reposition the abdominal organs to their physiological positions. The peritoneum and skin were closed with a single-layer continuous suture using 3 − 0 silk. The operation area was treated with povidone-iodine solution, then repeatedly wiped with 70% ethanol swabs two to three times before being covered and secured with a sterile dressing. Sham-operated mice underwent the same surgical procedure but without gallbladder removal. The surgical incision has healed well, with no local redness, swelling or discharge.

### RNA-Seq and differential expression analysis

Five liver tissue samples from each group were collected and the RNA-sequencing services were provided by Personal Biotechnology Co., Ltd. (Shanghai, China). Total RNA was isolated using the Trizol Reagent (Invitrogen Life Technologies), and the concentration, quality and integrity were then determined using a Nanodrop spectrophotometer (Thermo Scientific). Three micrograms of RNA was used as the input material for the RNA sample preparations. The libraries were then sequenced on an Illumina NovaSeq 6000 platform, generating 150 bp paired-end reads, the sequencing depth of data set is 6G. Total raw read data generated per sample ranged from 36.61 to 57.00 Mb. Initially, the raw data (i.e. raw reads) were processed using fastp^10^, with the objective of removing low-quality reads. This process yielded the clean reads. The clean reads were mapped to C57BL/6 mouse reference genome by STAR version 2.5. We used HTSeq statistics^11^ to compare the read count value of each gene as the original expression of the gene, and then used FPKM to standardise the expression. Principal Component Analysis reveals minor intra-group variations and significant inter-group differences. Differentially expressed genes(DEGs) were analysed by DESeq2^12^. DESeq2’s default p-value correction method employs the Benjamini-Hochberg (B-H) method to adjust p-values for controlling the false discovery rate (FDR). The column containing the adjusted p-values is named padj. Significant DEGs were identified depending on the criteria: FoldChange ≥ 1.5 and FDR < 0.05. Two-way hierarchical clustering was performed on the union set of differentially expressed genes (DEGs) using Euclidean distance and complete linkage. For visualization, gene expression values were row-wise Z-score normalized to compare relative expression patterns across samples, and the resulting heatmap was generated with the ComplexHeatmap R package. Kyoto Encyclopedia of Genes and Genomes (KEGG)^13^ pathway enrichment analysis was performed to identify biological pathways significantly overrepresented among the differentially expressed genes (DEGs). The analysis was conducted in R using the clusterProfiler package. All genes that were successfully mapped to KEGG pathways and detected in our RNA-seq experiment were used as the background/reference set for the analysis. A hypergeometric test (equivalent to a one-sided Fisher’s exact test) was applied to assess whether the observed overlap between the DEG list and each KEGG pathway gene set was statistically significant. To identify pathways broadly associated with the phenotypic change, the analysis was performed on the combined set of all DEGs (both up- and downregulated). The resulting p-values were adjusted for multiple testing using the Benjamini-Hochberg method, and pathways with a false discovery rate (FDR) adjusted p-value (q-value) of < 0.05 were considered statistically significantly enriched. Bioinformatic analysis was performed using the free online platform Personalbio GenesCloud (https://www.genescloud.cn).

In addition, Gene Set Enrichment Analysis (GSEA)^14^ was utilised to arrange all signature genes and RNA-seq data, as determined by the t-statistic of the differential expression analysis, according to their association with high or low expression of the core genes. The classification of gene sets as statistically significant was determined by the fulfilment of the following criteria: a nominal p-value less than 0.05, an absolute normalized enrichment score (NES) greater than 1, and false positive rate (FDR) q-value less than 0.25.

### Liquid chromatography–mass spectrometry-based metabolomics analysis

The liver tissue samples (25 mg ± 1 mg) of six replicates from each group were collected and mixed with beads and 500 µl of extraction solution (MeOH: ACN: H2O, 2:2:1 (v/v)). The extraction solution contains deuterated internal standards^15^. Then the mixed samples were homogenized (35 Hz,4 min) and sonicated for 5 min in 4 ℃ water bath, the step repeat for three times. The samples were incubated for 1 h at -40 ℃ to precipitate proteins, after which they were centrifuged at 4 °C for 15 min at 12,000 rpm and the supernatant was collected for analysis. The quality control (QC) sample was prepared by mixing an equal aliquot of the supernatant of samples. Transfer the supernatant to a sample vial for testing. Combine equal volumes of supernatant from all samples to create a QC sample for testing. LC-MS/MS analyses were performed using a UHPLC system (Vanquish, Thermo Fisher Scientific) with a Phenomenex Kinetex C18 (2.1 mm × 50 mm, 1.7 μm) coupled to an Orbitrap Exploris 120 mass spectrometer (Thermo Fisher Scientific). The mobile phase A was an aqueous solution containing 25 mmol/L of both ammonium acetate and ammonia, while phase B is acetonitrile; The temperature of the auto-sampler was set at 4 °C, and the injection volume was measured at 2 µL(Wang J et al. Metabolomics, 2016, 12(7):116). Primary and secondary mass spectrometry data was acquired by the Orbitrap Exploris 120 mass spectrometer under the control of Xcalibur software (Version 4.4, Thermo). Detailed parameters are as follows: Sheath gas flow rate: 50 ArP, Auxiliary gas flow rate: 15 ArP, Capillary temperature: 320 °C, Full MS resolution: 60,000, MS/MS resolution: 15,000, Collision energy: SNCE 20/30/40, Spray voltage: 3.8 kV (positive) or -3.4 kV (negative). The raw data were converted to the mzXML format using ProteoWizard and subsequently processed for peak detection, extraction, alignment, and integration using XChromatography Mass Spectrometry (XCMS). Subsequently, the BiotreeDB database was employed for the purpose of metabolite annotation^16^. The raw data contains 3 quality control (QC) samples and 18 experimental samples, from which 46,964 features were extracted. The subsequent analysis involved the utilisation of a bespoke R package for the purpose of visualisation. The stability of the detection can be determined by the variation in the response peak height of the internal standard within QC samples.

To minimize the impact of detection system errors and better highlight biological significance, we performed a series of data preparation and organization steps. The following procedures were included:①Outlier filtering: Filtered individual features to remove noise. Outliers were filtered based on relative standard deviation (RSD, i.e., coefficient of variation, CV); ②Missing value filtering: Filtered individual features. Retained peak area data only if no more than 50% of a single group or 50% across all groups contained missing values; ③Missing value imputation: Simulated missing values in raw data (missing value recoding). The numerical simulation method involved imputation using half the minimum value;④Data Normalization: Normalization using an internal standard (IS)^17^. Background peaks were removed if the intensity in the procedure blank sample was ≥ 0.3 fold of that in the biological samples. After preprocessing, 43,937 features were retained. Among these, 2,272 were secondary qualitative compounds. The final dataset, which contained information on peak number, sample name, and normalised peak area, was imported into SIMCA18.0.1 software package (Sartorius Stedim Data Analytics AB, Umea, Sweden) for multivariate analysis. The data were scaled and log-transformed to minimise the impact of noise and high variability of the variables. After these changes, PCA (principal component analysis, PCA) was used to reduce the number of variables in the data. We used a 95% confidence interval in the PCA score plot to identify potential outliers in the dataset.

The metabolite orthogonal partial least squares discriminant analysis (OPLS-DA) was performed in order to visualise the discrimination between cases and controls, as well as to calculate VIP (variable importance in the projection) values for the purpose of prioritising differential metabolites.

The metabolite intensity data were subjected to log-transformation and Pareto-scaling, and subsequently modelled with group classification as the Y variable. The OPLS-DA model was constructed with one predictive component and orthogonal components determined by cross-validation. The performance of the system was evaluated by employing the R² and Q² values, with the outcomes of this evaluation being validated through the implementation of permutation testing. Metabolites were identified using an in-house R package and annotated using the HMDB. The identification results reference the Metabolomics Standards Initiative (MSI) for annotating metabolite identification levels: Level 1: The metabolite in the sample matches the standard at MS1, MS2, and RT; Level 2: The metabolite in the sample matches both MS1 and MS2 in public databases; Level 3: The metabolite in the sample matches the compound in MS1, MS2, and RT; Level 4: Unknown compound^18^.The Student’s t test method was used to calculate the difference between groups based on the metabolite standardized expression data, and the multiple test P values were corrected by the FDR method^19^.

Variables with VIP score > 1.0 were identified as significant contributors to group separation. Multivariate statistics were utilised to identify differential metabolites, where a P value < 0.05 from the student’s t-test, VIP score > 1, Fold Change ≥ 1.5 were selected as significant differentially expressed metabolites (DEMs)^20^. The abundance data of these metabolites were log10-transformed and row-wise Z-score normalized. A two-way hierarchical clustering heatmap was generated using Pearson correlation distance and Ward’s linkage method. KEGG pathway enrichment analysis was performed using the MetaboAnalyst web server. The hypergeometric test was applied, using all annotated metabolites from the experiment as the reference background. Significance was set at p value < 0.05 (the corresponding FDR q-values are also presented).

A correlation analysis should be performed between DEGs (FoldChange ≥ 1.5 and FDR < 0.05) and DEMs (VIP score > 1, P value < 0.05, Fold Change ≥ 1.5) using the `cor` program in R in order to calculate Pearson’s correlation coefficients between genes and metabolites. It is evident that the differential group demonstrates a higher number of correlations between genes and metabolites. Consequently, it is recommended that the top 100 correlation pairs are selected in order to generate a correlation coefficient clustering heatmap and a correlation network diagram. Correlation analysis was performed using the OmicShare tool, a free online platform for data analysis (https://www.omicshare.com/tools). Concurrently, the mapping of DEGs (FoldChange ≥ 1.5 and FDR < 0.05) and DEMs (VIP score > 1, P value < 0.05, Fold Change ≥ 1.5) to the KEGG pathway database was undertaken, with the objective of obtaining shared pathway information^21^. The metabolic pathway analysis results of differentially expressed metabolites and their associated transcripts were then visualised using the R language pathview package.

## Results

### Transcriptome and DEGs analysis of liver on the seventh day post-cholecystectomy

**Fig. 1:**
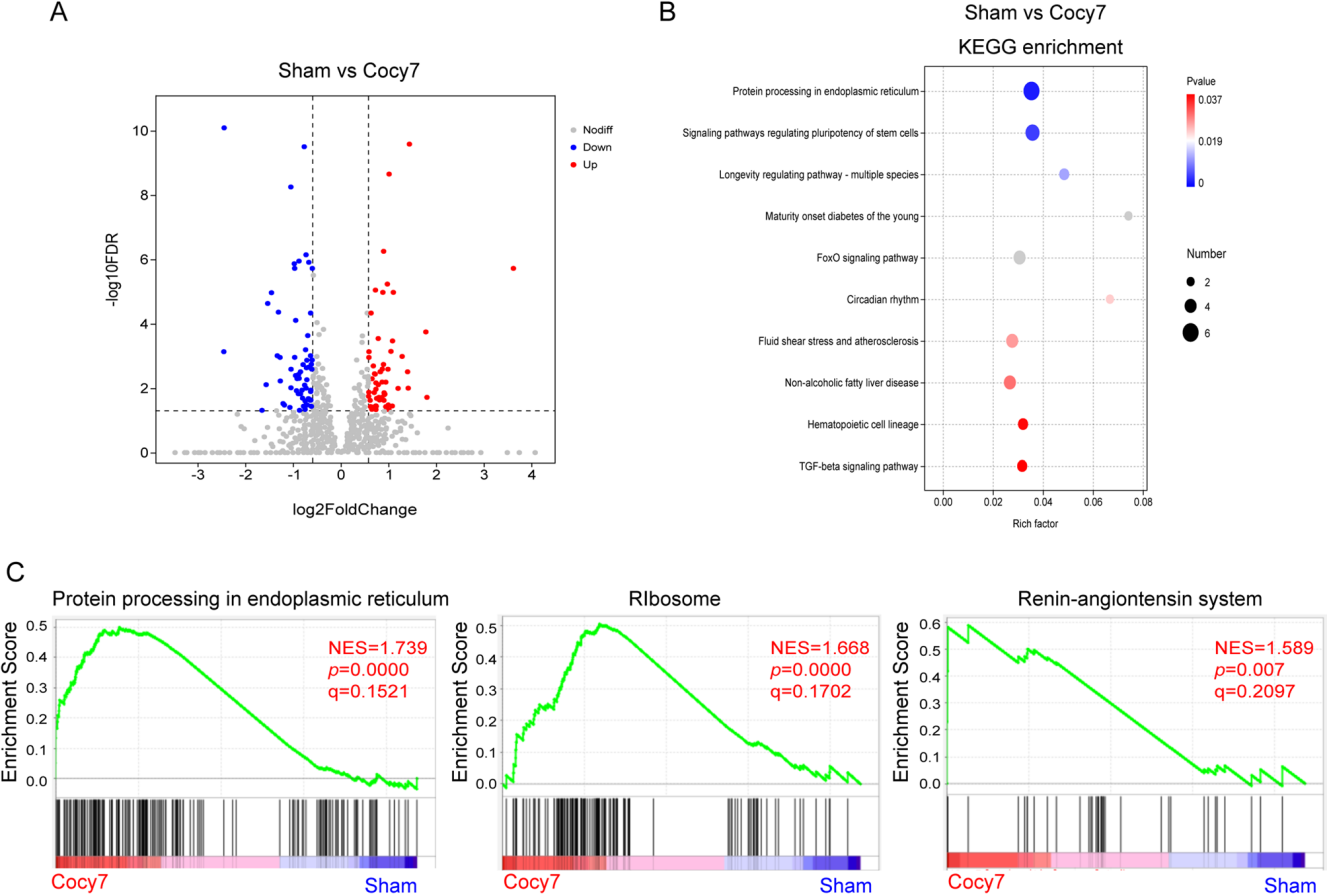
The differential expression of genes in the liver of mice on the seventh day following cholecystectomy and sham operation. (**A**) Volcano plots were generated to illustrate the distribution of FDR values [-log10(FDR value)] and fold change values [log2 (fold change)] of differentially expressed genes (DEGs). As illustrated, downregulated genes are indicated by blue colouration, whilst those which are upregulated are denoted by red (FDR < 0.05 and Fold Change ≥ 1.5)..(**B**) KEGG analysis of DEGs (*p* < 0.05). (**C**) GSEA of RNA-seq data from liver tissue on the seventh day following either cholecystectomy or sham operation. Cocy7, liver tissue on the 7th day after cholecystectomy; Sham, liver tissue on the 7th day after sham operation.

In order to elucidate the transcriptional modulation of the liver after cholecystectomy, transcriptomic analysis was carried out on the liver of mice on the seventh and thirtieth day post-cholecystectomy, as well as on the liver on the seventh day after the sham operation (denoted by Cocy7, Cocy30 and Sham, respectively). Principal component analysis reveals minor variations within groups and significant differences between them (Supplementary Fig. 1A). Applying the cutoffs of fold change ≥ 1.5 and FDR < 0.05 for the filtering of differentially expressed genes (DEGs), the results demonstrate that 59 genes are up-regulated and 63 genes are down-regulated in the Cocy7 group (Supplementary table A-1). Volcano plots provide a highly visual means of comprehensively representing the distribution of differentially expressed genes across various groups. In addition to the distribution, these plots furnish the reader with information regarding the statistical significance and magnitude of the fold change in gene expression (Fig. 1A). KEGG enrichment analysis of 122 DEGs in the Cocy7 group revealed that they gathered into the top 10 pathway terms (raw *p* < 0.05, adjusted *P* > 0.05) (Fig. 1B) (Supplementary Table A-2). It was suggested that DEGs may be correlated with the protein processing in the endoplasmic reticulum and FoxO signaling pathways.

In order to achieve a more precise reflection of the overall state of activation or repression of the gene sets regulated after cholecystectomy, GSEA was performed. The findings of the study demonstrated a positive correlation between cholecystectomy and the top three signatures of Protein Processing in the Endoplasmic Reticulum, Ribosome, and Renin-Angiotensin System in the Cocy7 group (Fig. 1C) (Supplementary table A-3). The processing of proteins in the endoplasmic reticulum (ER) refers to the process undergone by newly synthesised polypeptide chains in eukaryotes. Following synthesis, these chains enter the ER, where they undergo post-translational modifications, including glycosylation and disulfide bond formation. The resultant multi-subunit complexes fold and assemble within the ER, and are subsequently transported to the Golgi apparatus^22^. Ribosomes are the cellular factories responsible for protein production. The process of ribosome biogenesis in eukaryotes is a highly complex and precise mechanism that entails the synthesis and accurate assembly of four rRNAs and approximately 80 ribosomal proteins^23,24^. These results indicated that protein synthesis and processing in the liver are activated in the early stages following cholecystectomy. The renin–angiotensin system (RAS) is a complex network comprising circulating molecules, enzymes and receptors, which are expressed in multiple organs. The liver, the primary source of angiotensinogen for central RAS in the circulation, produces and secretes angiotensinogen, which serves as the precursor for the angiotensins^25^. The angiotensins are the active peptides that constitute the renin-angiotensin system. The role of local RAS in the liver has been demonstrated to be particularly related to the process of regeneration in cases of liver fibrosis, injury and apoptosis^26^.

### Transcriptome and DEGs analysis of liver on the thirtieth day post-cholecystectomy

**Fig. 2:**
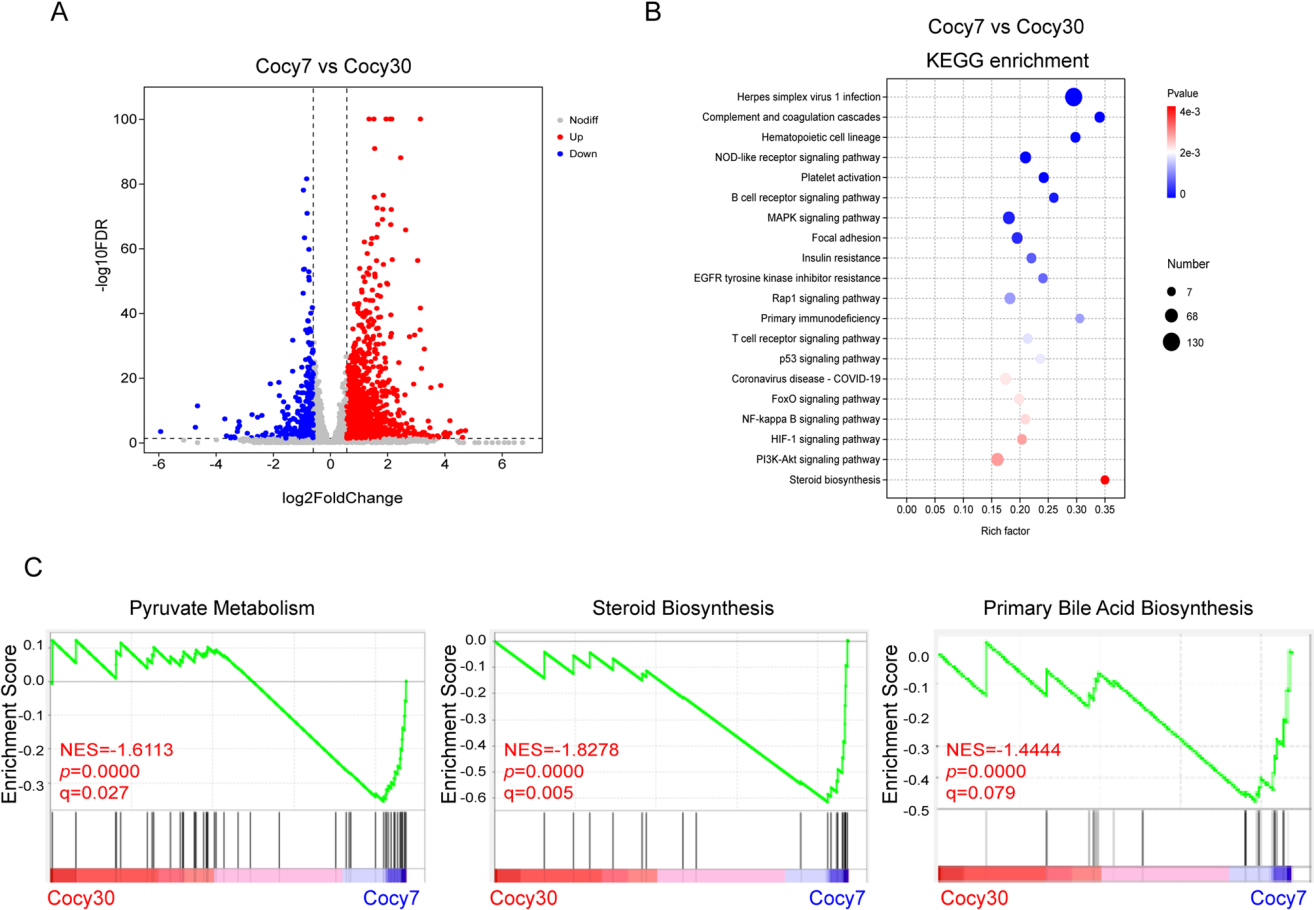
The differential expression of genes in the liver of mice on the thirtieth day and seventh day following cholecystectomy. (**A**) The distribution of FDR values [-log10(FDR value)] and fold change values [log2 (fold change)] of DEGs was illustrated by volcano plots. As demonstrated in the figure, downregulated genes are indicated by blue colouration, whilst those that are upregulated are denoted by red (FDR < 0.05 and Fold Change ≥ 1.5). (**B**) KEGG analysis of DEGs (*p* < 0.05). (**C**) GSEA of RNA-seq data from liver tissue on the thirtieth day and seventh day following cholecystectomy. Cocy30, liver tissue on the 30th day after cholecystectomy; Cocy7, liver tissue on the 7th day after cholecystectomy.

As time progresses, with the 30th day following the cholecystectomy operation, the data showed that 1806 genes are up-regulated and 465 genes are down-regulated in the Cocy30 group in comparison with the Cocy7 group (Fold Change ≥ 1.5 and FDR < 0.05 as cutoffs) (Fig. 2A) (Supplementary Table B-1). KEGG enrichment analysis of 2271 DEGs in the Cocy30 group revealed that they clustered into the top 20 significant pathway terms (raw *p* < 0.05, adjusted *P* < 0.05) (Fig. 2B) (Supplementary Table B-2). Of particular note is the significant correlation of the DEGs with complement and coagulation cascades, hematopoietic cell lineage and NOD-like receptor signaling pathway. Both hematopoietic cell lineage and complement and coagulation cascades are immune-related pathways^27,28^. The NOD-like receptor signaling pathway is classified as a member of a distinct family of pattern recognition receptors. These receptors are responsible for the detection of a variety of pathogens and the generation of innate immune responses. GSEA revealed a negative correlation between gallbladder removal and the top four signatures of pyruvate metabolism, steroid and primary bile acid biosynthesis, citrate cycle in the Cocy30 group (Fig. 2C) (Supplementary Table B-3). These data indicated that mitochondrial energy metabolism and cholesterol catabolism in the liver are suppressed by the late stage of cholecystectomy.

**Fig. 3:**
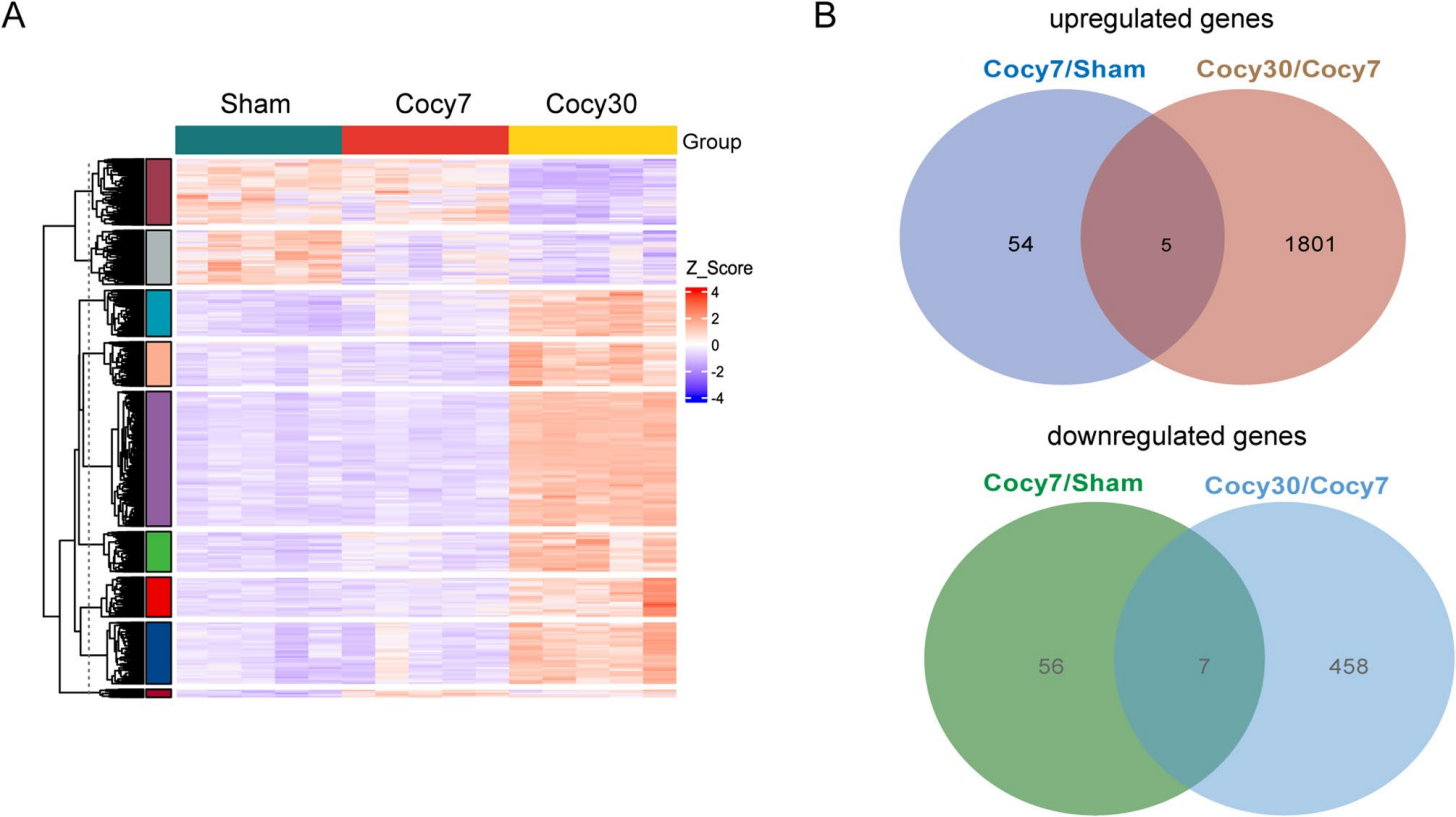
Gene expression profiles of liver in three groups after cholecystectomy. (**A**) The heat map of all the DEGs in the liver of mice on the thirtieth day, seventh day following cholecystectomy and sham groups (*n* = 5) (Z-Score Standardization). (**B**) Venn diagram showing the number of upregulated and downregulated genes between the indicated groups. Cocy30, liver tissue on the 30th day after cholecystectomy. Cocy7, liver tissue on the 7th day after cholecystectomy; Sham, liver tissue on the 7th day after sham operation.

In summary, gene expression profiles among the three groups showed that Cocy30 group exhibited highly significant disparities in gene expression compared with Cocy7 and Sham groups (Fig. 3A). As the duration of the cholecystectomy increased, five genes (Clic3, Utp14b, Tfrc, Pcdh12 and Mme) were found to be consistently overexpressed. Concurrently, 54 genes ceased to be overexpressed, while 1,801 genes began to be overexpressed. Meanwhile, a total of seven genes (Fkbp5, Fbxo31, Tacc2, 2010003K11Rik, Arhgef37, Il1r1 and Slc8b1) were found to be consistently downregulated, while 56 genes ceased downregulation and 458 genes began to downregulate (Fig. 3B). These results suggested that Cholecystectomy could induce immune activation and impair cholesterol metabolism in the liver.

### Profiling and bioinformatics analysis of the hepatic metabolome on day 7 post-cholecystectomy

In mice subjected to cholecystectomy, there was an increase in both basal metabolic rate and hepatic triglyceride content^6,7^. In this study, we sought to elucidate the alterations in the liver metabolome of cholecystectomised mice in comparison with those of sham-operated mice. We analyzed the liver metabolome in randomly selected Cocy7, Cocy30 and Sham mice (*n* = 6). To test the quality of metabolome data, principal component analysis (PCA) was conducted using the metabolic profiles of the different groups. PC1 explains 28.13% of the variance in metabolomics, and PC2 explains 18.1% using integral metabolomics data (Supplementary Fig. 1B). The OPLS-DA and permutation intercept results showed that the samples are all contained within the specified confidence interval, thereby substantiating the reliability of the collected data (Supplementary Fig. 1C&D).

**Fig. 4:**
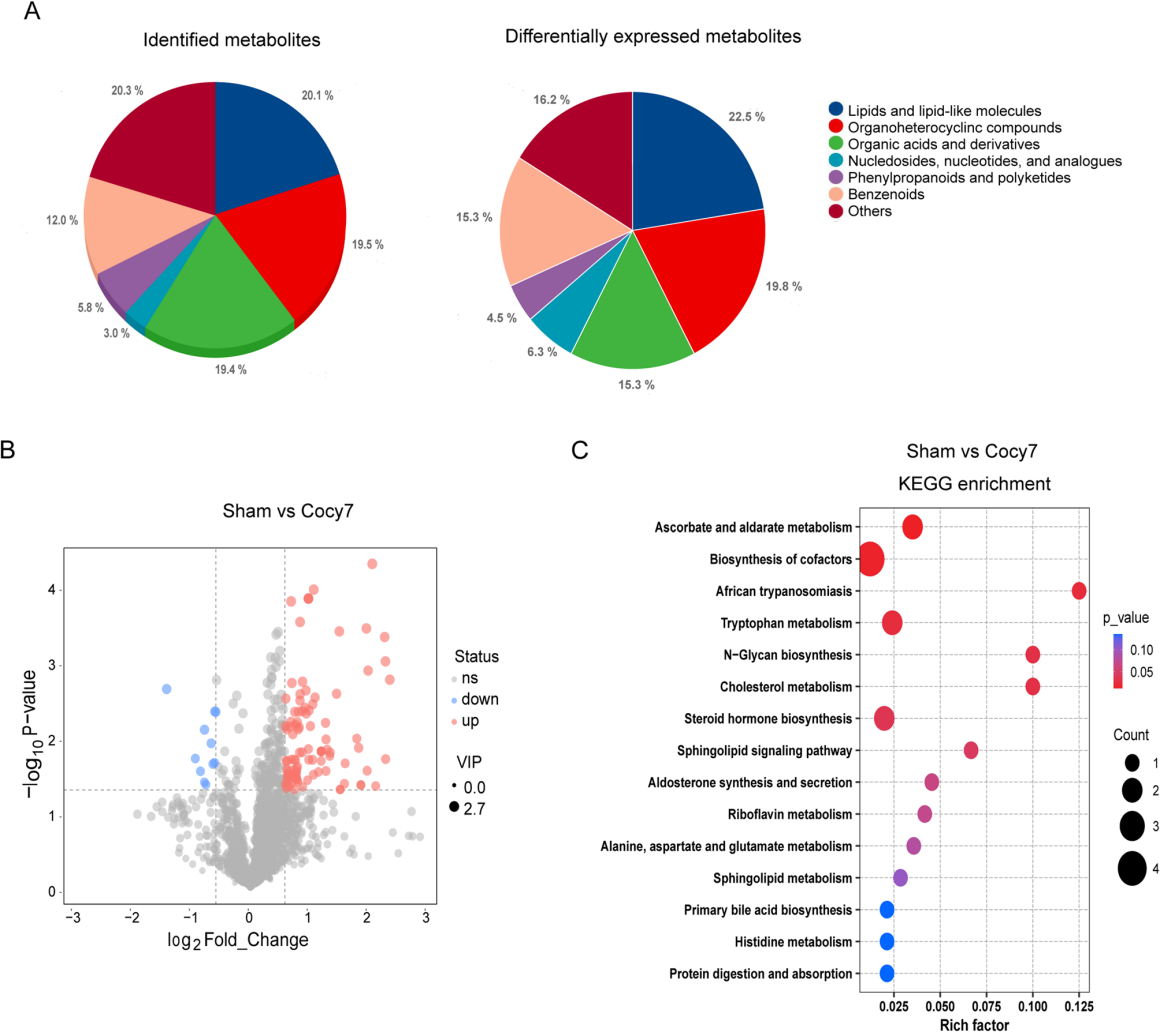
Metabolic profiling and altered metabolism-related pathways of liver tissue on the seventh day following cholecystectomy. (**A**) Pie graph of identified metabolite class composition and (**B**) Significant differentially expressed metabolites (DEMs) in the Cocy7 male mice (VIP score > 1, P value < 0.05 and Fold Change ≥ 1.5 as cutoffs). (**C**) Volcano plots of DEMs in the Cocy7/Sham groups. Blue denotes down-regulated metabolites, and red represents up-regulated metabolites (*n* = 6). (**D**) Bubble illustration of top15 ranked enriched KEGG pathway terms. The diameter of the solid circle denotes the number of DEMs enriched and the color showing the *p*-value in the corresponding pathway. Cocy7, liver tissue on the 7th day after cholecystectomy; Sham, liver tissue on the 7th day after sham operation.

This investigation resulted in the identification of a total of 2,272 metabolites, including 1,335 in positive ion mode and 937 in negative ion mode (Supplementary Table C-1). Of the 2,272 metabolites identified, 111 were found to be significantly differentially expressed (VIP score > 1, P value < 0.05 and Fold Change ≥ 1.5) in the Cocy7 group, compared with the Sham group (Supplementary Table C-1). 100 of which were upregulated and 11 downregulated (Supplementary Table C-1). The identified and differentially expressed metabolites (DEMs) were classified into several superclasses, including lipids and lipid-like molecules, organoheterocyclic compounds, benzenoids, organic acids and derivatives, and nucleotides (Fig. 4A). In this study, the top two significantly altered superclasses in the Cocy7 group were found to be lipid metabolites (*n* = 25) and organoheterocyclic compounds (*n* = 22). It is noteworthy that the vast majority of lipid metabolites and organoheterocyclic compounds in the Cocy7 group exhibited an increase in expression. The downregulated DEMs included N-Acetylhistamine, 3-Amino-6-methyl-1-[3-(trifluoromethyl)phenyl]pyridazin-4-one, (E)-1-Hydroxy-2-methylbut-2-enyl 4-diphosphate, Mofebutazone and Noscapine.

The KEGG enrichment analysis of the DEMs showed that the top 3 pathways such as ascorbate and aldarate metabolism, biosynthesis of cofactors and tryptophan metabolism were significantly enriched in the Cocy7 group (Fig. 4D) (Supplementary Table C-2). Secondly, both cholesterol metabolism and steroid hormone biosynthesis pathway were significantly altered. The collective analysis of the results indicated that there was a disturbance in the homeostasis of lipids and organoheterocyclic metabolites in the Cocy7 group.

### Profiling and bioinformatics analysis of the hepatic metabolome on day 30 post-cholecystectomy

**Fig. 5:**
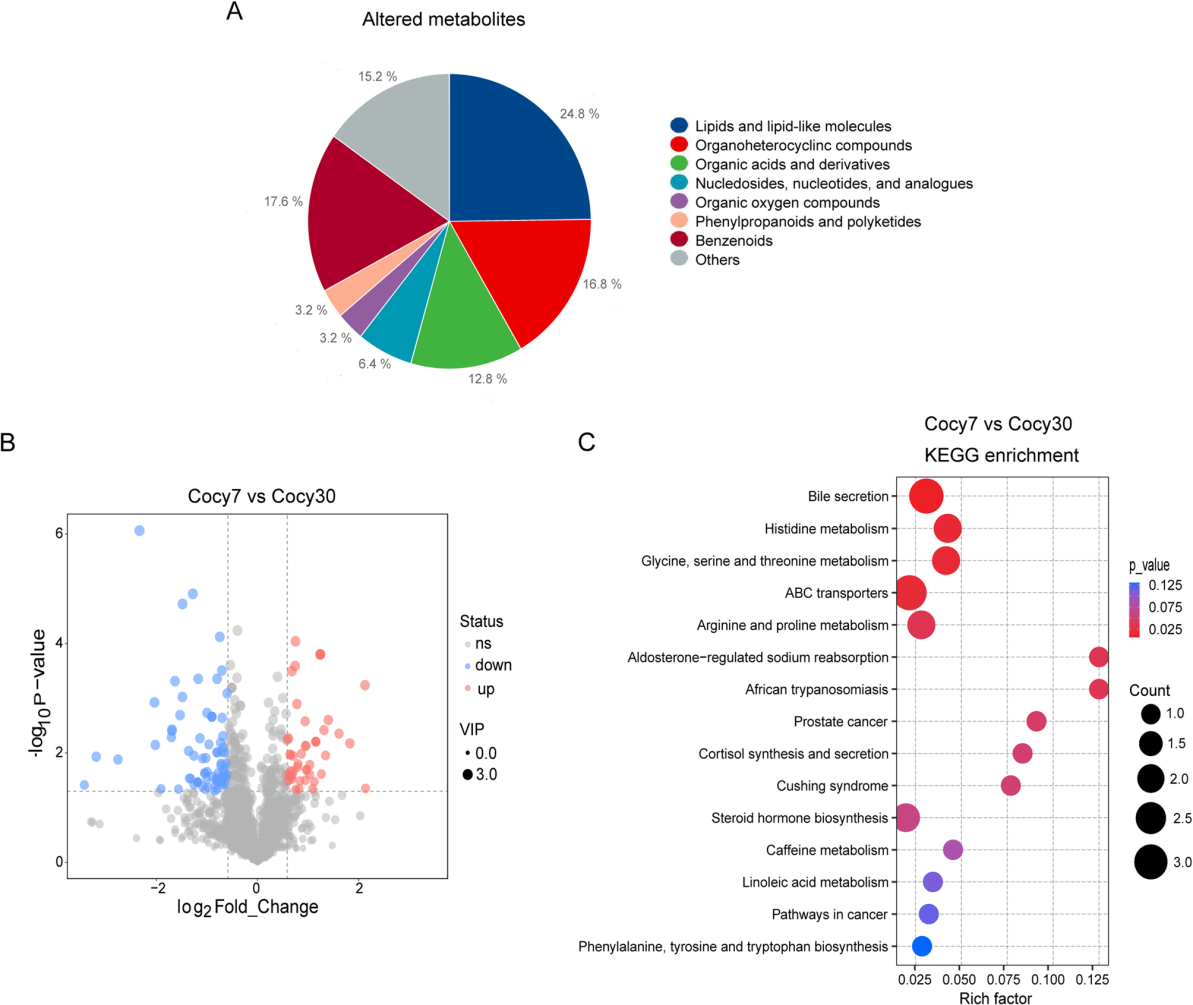
Metabolic profiling and altered metabolism-related pathways of liver tissue on the thirtieth day following cholecystectomy. (**A**) Significant differentially expressed metabolites (DEMs) in the Cocy30 male mice (VIP score > 1, P value < 0.05 and Fold Change ≥ 1.5 as cutoffs). (**B**) Volcano plots of DEMs in the Cocy30/Cocy7 groups. Blue denotes down-regulated metabolites, and red represents up-regulated metabolites (*n* = 6). (**C**) Bubble illustration of top15 ranked enriched KEGG pathway terms. The diameter of the solid circle denotes the number of DEMs enriched and the color showing the *p*-value in the corresponding pathway. Cocy30, liver tissue on the 30th day after cholecystectomy; Cocy7, liver tissue on the 7th day after cholecystectomy.

In comparison with the Cocy7 group, 125 metabolites were identified as being differentially abundant (VIP score > 1, P value < 0.05 and Fold Change ≥ 1.5) in the Cocy30 group, 76 of which were found to be overexpressed and 49 underexpressed (Supplementary Table D-1). The DEMs were then categorised into a series of overarching classifications, namely lipids and lipid-like molecules, organoheterocyclic compounds, benzenoids, organic acids and derivatives, organic oxygen compounds, and finally nucleotide compounds. Of these, lipid metabolites (*n* = 31), benzenoids (*n* = 22) and organoheterocyclic compounds (*n* = 21) accounted for the top 3 largest proportion of significantly altered metabolites in the Cocy30 group (Fig. 5A). Secondly, the primary element of organic acids and derivatives (*n* = 16) pertains to amino acids, peptides, and analogues(*n* = 10). The identified DEMs are shown in Fig. 5B. Notably, most of lipid metabolites in the Cocy30 group were upregulated. Upregulated DEMs included cortisol, 21-deoxycortisol, isodeoxycholic acid, ursodeoxycholic acid, deoxycholic acid and others. KEGG pathway enrichment analysis of the DEMs indicated that bile secretion, histidine metabolism and glycine, serine and threonine metabolism was the most significantly enriched in the Cocy30 group (Fig. 5C) (Supplementary Table D-2). Secondly, there was a significant alteration in both ABC transporters and cortisol synthesis and secretion pathways. ATP-binding cassette (ABC) transporters are a major class of sterol exporters. They are responsible for both cholesterol efflux from peripheral cells and the elimination of excess cholesterol and dietary sterols^29,30^. Human subfamily-G transporters are predominantly associated with lipid and sterol metabolism, including ABCG1/4/5/8. It has been established that ABCG1 and ABCG4 play a pivotal role in the mediation of cholesterol trafficking within the plasma membrane and endosomes and were believed to regulate cholesterol homeostasis within the macrophage-rich tissues^31,32^. ABCG5 and ABCG8 function as a heterodimeric complex (ABCG5/G8) and are responsible for biliary and transintestinal secretion of cholesterol and dietary sterols^33^. Combined, the findings of this study indicated that the homeostasis of lipid metabolism, with a particular emphasis on cholesterol metabolism, was compromised in the Cocy30 group.

**Fig. 6:**
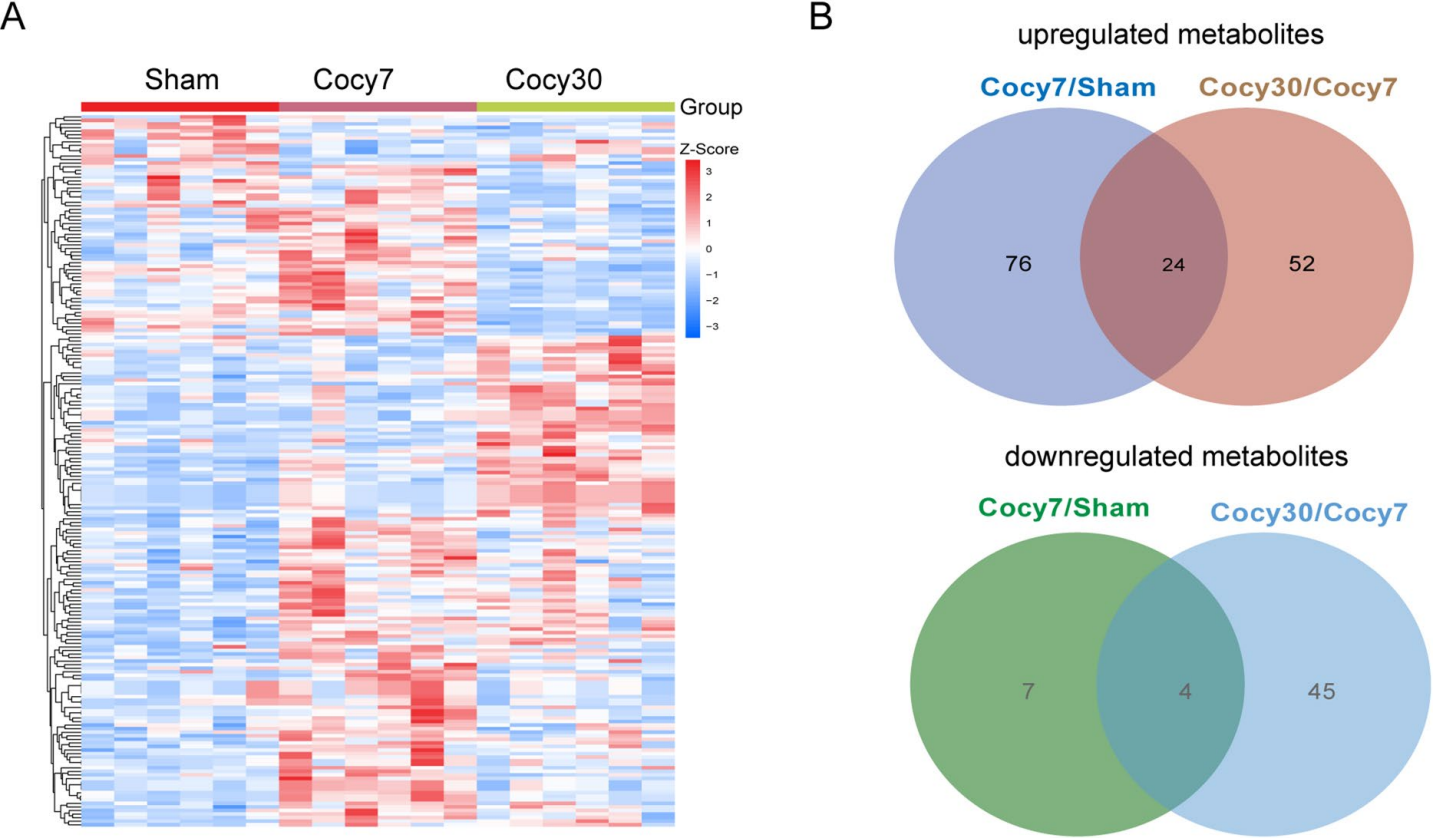
Profiles of differentially expressed metabolites in the liver of cholecystectomised and sham groups. (**A**) The heatmap showed the relative contents of DEMs (*n* = 6). The abundance data for these metabolites were log₁₀-transformed and Z-score normalised on a row-by-row basis. A two-way hierarchical clustering heatmap was then generated using the Ward’s linkage method and Pearson correlation distance. (**B**) Venn diagram showing the number of upregulated and downregulated DEMs between the indicated groups. Cocy30, liver tissue on the 30th day after cholecystectomy; Cocy7, liver tissue on the 7th day after cholecystectomy; Sham, liver tissue on the 7th day after sham operation.

Vertically, the results demonstrated that there were significant differences in the metabolism expression profiles among the three groups. The Cocy7 group exhibited elevated levels of metabolites in comparison to the Sham group, while the Cocy30 group demonstrated metabolic reprogramming, with the number of upregulated metabolites exhibiting a slight increase over that of downregulated metabolites compared to the Cocy7 group (Fig. 6A). An analysis of metabolite expression during cholecystectomy revealed a total of 24 metabolites that exhibited consistent upregulation. Concurrently, 4 metabolites demonstrated consistent downregulation (Fig. 6B) (Table 1).

**Table 1:** The metabolites list exhibiting sustained deregulation over time.

No.	MS2 name	HMDB	KEGG	Super.Class	Sub.Class	
The metabolites exhibiting sustained upregulation(24).						
1	Octocrylene	HMDB0255910		Benzenoids	Diphenylmethanes	
2	2-Pyrrolidinone, 5-[(1E,3R)-4,4-difluoro-3-hydroxy-4-phenyl-1-buten-1-yl]-1-[6-(2 H-tetrazol-5-yl)hexyl]-, (5R)-			Benzenoids	Benzene and substituted derivatives	
3	21-Deoxycortisol	HMDB0004030	C05497	Lipids and lipid-like molecules	Pregnane steroids	
4	Glycolithocholic acid 3-sulfate	HMDB0002639	C11301	Lipids and lipid-like molecules	Bile acids, alcohols and derivatives	
5	10-Hydroxy-2,4b,8-trimethyl-2-(2-((3,4,5-trihydroxy-6-(hydroxymethyl)tetrahydro-2 H-pyran-2-yl)oxy)ethyl)dodecahydro-4a,10a-epoxyphenanthrene-8-carboxylic acid			Lipids and lipid-like molecules	Fatty acyl glycosides	
6	5-Heptenoic acid, 7-[(1R,2R,3 S,5 S)-2-[(1E,3R)-3-(2,3-dihydro-1 H-inden-2-yl)-3-hydroxy-1-propen-1-yl]-3-fluoro-5-hydroxycyclopentyl]-, (5Z)-			Lipids and lipid-like molecules	Fatty acids and conjugates	
7	Capsianoside_V	HMDB0030737		Lipids and lipid-like molecules	Terpene glycosides	
8	Lithocholic acid 3-sulfate	HMDB0000907		Lipids and lipid-like molecules	Bile acids, alcohols and derivatives	
9	Tetradecanedioic acid	HMDB0000872		Lipids and lipid-like molecules	Fatty acids and conjugates	
10	2,7-Dihydroxy-8-(5-hydroxy-4,8-dimethyl-7-methylidene-3-oxononyl)-4a,7-dimethyl-1,2,3,4,5,6,8,9,10,10a-decahydrophenanthrene-1-carboxylic acid			Lipids and lipid-like molecules	Steroid acids	
11	Cholic acid/Muricholic acid, tryptophan-conjugated			Lipids and lipid-like molecules	Bile acids, alcohols and derivatives	
12	Ribothymidine	HMDB0000884		Nucleosides, nucleotides, and analogues	Pyrimidine nucleosides	
13	1-Methylpseudouridine			Nucleosides, nucleotides, and analogues	Nucleoside and nucleotide analogues	
14	3-Methyluridine	HMDB0004813		Nucleosides, nucleotides, and analogues	Pyrimidine nucleosides	
15	Allopurinol-1-ribonucleoside	HMDB0000481		Nucleosides, nucleotides, and analogues	Pyrazolo[3,4-d]pyrimidine glycosides	
16	2-(5,6-Dihydroxy-3-methoxycarbonylcyclohex-3-en-1-yl)oxypropanoic acid			Organic acids and derivatives	Dicarboxylic acids and derivatives	
17	Kynurenine	HMDB0000684	C00328	Organic oxygen compounds	Carbonyl compounds	
18	2-(3-Phenyl-1 H-1,2,4-triazol-5-yl)pyridine			Organoheterocyclic compounds	Pyridyltriazoles	
19	Isopentenyladenine	HMDB0245646	C04083	Organoheterocyclic compounds	Purines and purine derivatives	
20	Pentadecanedioylcarnitine			others	Others	
21	Tetradecadienoylcarnitine (Car(14:2))	HMDB0241377		others	Others	
22	D-myo-Inositol, 1-[2-hydroxy-3-[(1-oxo-9,12-octadecadienyl)oxy]propyl hydrogen phosphate], [S-(Z, Z)]-			others	Others	
23	4-propylidenepyrrolidine-2-carboxy-adenylate			others	Others	
24	Lanceolatin B			Phenylpropanoids and polyketides	Flavones	
The metabolites exhibiting sustained downregulation(4)						
1	4,4,6-Trimethyl-1-(1-naphthyl)-1,4-dihydro-2-pyrimidinethiol			Benzenoids	Naphthalenes	
2	Fenfluramine	HMDB0252200	C06996	Benzenoids	Phenethylamines	
3	Mofebutazone	HMDB0254828		Benzenoids	Benzene and substituted derivatives	
4	N-Acetylhistamine	HMDB0013253	C05135	Organic acids and derivatives	Carboxylic acid derivatives	

### Integrative analysis of transcriptome and metabolome in the liver with cholecystectomy

**Fig. 7:**
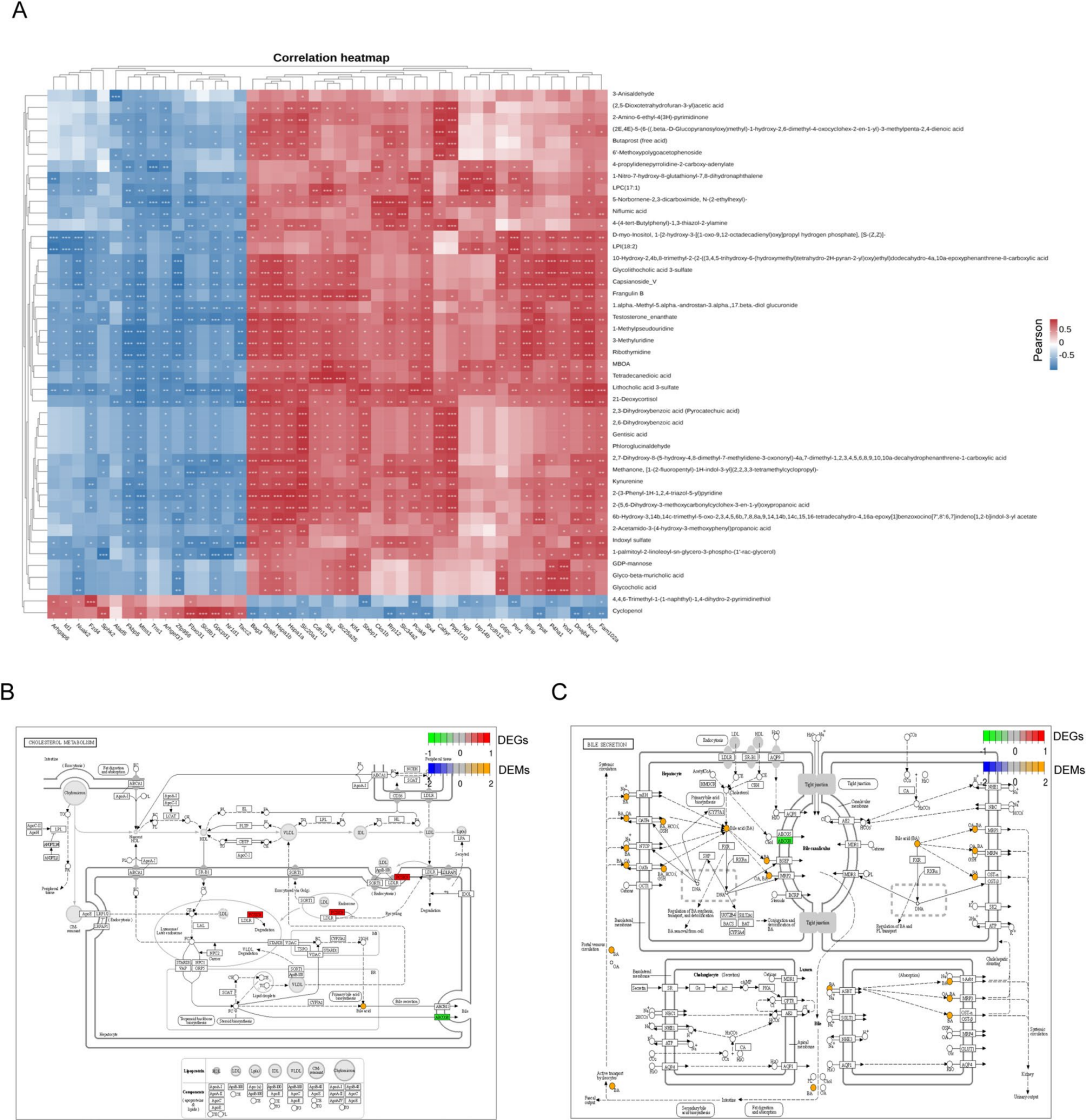
Integrative analysis of transcriptome and metabolome in the liver of mice on the seventh day following cholecystectomy. (**A**) Correlation heatmap of links between top 100 differentially expressed genes and metabolite pairs based on the Pearson correlation algorithm. The color bar denotes the value of correlation coefficient. *p < 0.05, **p < 0.01, ***p < 0.001. (**B**) DEGs and DEMs in cholesterol metabolism and bile secretion(C). The levels of differential expression of metabolites are indicated from low to high using shades of blue to yellow. The levels of differential expression of transcripts are indicated from low to high using shades of green to red.

**Fig. 8:**
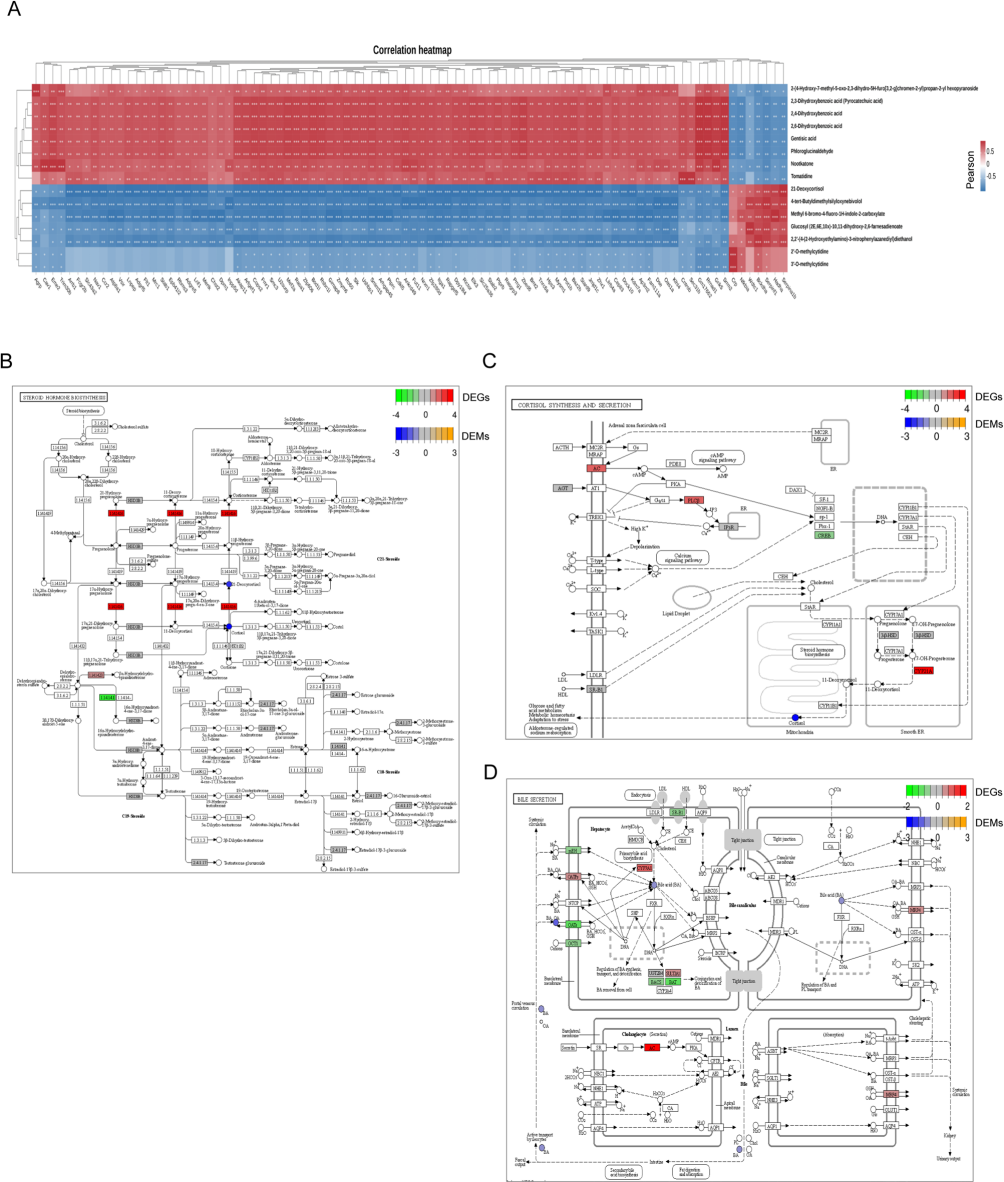
Integrative analysis of transcriptome and metabolome in the liver of mice on the thirtieth day and seventh day following cholecystectomy. (**A**) Correlation heatmap of links between top 100 differentially expressed genes and altered metabolite pairs based on the Pearson correlation algorithm. The colour bar denotes the value of correlation coefficient. *p < 0.05, **p < 0.01, ***p < 0.001. (**B**) DEGs and DEMs in steroid hormone biosynthesis, cortisol synthesis and secretion (**C**) and bile secretion(D). The levels of differential expression of metabolites are indicated from low to high using shades of blue to yellow. The levels of differential expression of transcripts are indicated from low to high using shades of green to red.

A core genes-metabolites regulation network must decipher the significant clustered genes and metabolites responsible for the changes following gallbladder removal. Comparing the RNA-seq and metabolomic data, we built a composite chart of DEGs and DEMs, which were simultaneously enriched in the same pathways. A correlation analysis was conducted, which demonstrated that the differential group exhibited a higher number of correlations between genes and metabolites. The top 100 correlation pairs were selected for further analysis, with the objective of generating a correlation coefficient clustering heatmap in the sham vs. Cocy7 comparison (Fig. 7A) (Supplementary Table E-1) and Cocy7 vs. Cocy30 comparison (Fig. 8A) (Supplementary Table E-2). Subsequent analysis involved the integration of DEGs and DEMs into the KEGG pathway database, with the objective being the procurement of shared pathway information. In the sham vs. Cocy7 comparison, several pathways were identified, including Amino Sugar and Nucleotide Sugar Metabolism (ko00520), Purine Metabolism (ko00230), Alanine, Aspartate and Glutamate Metabolism (ko00250), Bile Secretion (ko04276), Protein Digestion and Absorption (ko04974), and Cholesterol Metabolism (ko04979). A number of important pathways have been identified between Cocy7 and Cocy30, including phenylalanine metabolism (ko00330), arginine and proline metabolism (ko00360), steroid hormone biosynthesis (ko00140), ABC transporters (ko02010), cortisol synthesis and secretion (ko04927), bile secretion. Both comparisons contained cholesterol metabolism, cortisol synthesis and secretion, steroid hormone biosynthesis, ABC transporters, and Bile Secretion. These pathways form a complete metabolic network centred around the cholesterol molecule, which has been designed according to the biological logic of “acquisition-utilization-clearance”. Furthermore, we analysed DEGs and DEMs implicated in cholesterol metabolism (Fig. 7B) and bile secretion (Fig. 7C) in the Sham vs. Cocy7 comparison, as well as steroid hormone biosynthesis (Fig. 8B), cortisol synthesis and secretion (Fig. 8C), and bile secretion (Fig. 8D) in the Cocy7 vs. Cocy30 comparison. The results of the integrative analysis showed that liver metabolic abnormalities centred on cholesterol may represent the most significant impact of cholecystectomy.

## Discussion

Gallstone disease is a prevalent condition, and cholecystectomy is a frequently performed surgical procedure. A preceding survey on the correlation between non-alcoholic fatty liver disease (NAFLD) and cholecystectomy has indicated that cholecystectomy itself may be a risk factor for NAFLD. Cortes et al. discovered that hepatic fat content, serum insulin and apolipoprotein B (ApoB) levels had increased significantly two years after cholecystectomy^34^. In accordance with the findings observed in human subjects, serum and hepatic triglyceride (TG) concentrations, as well as hepatic very-low-density lipoprotein (VLDL)-TG, were all found to be significantly elevated in C57BL/6 mice two months following gallbladder removal^7^. The potential correlation between cholecystectomy and NAFLD remain to be fully elucidated. This study aims to analyse the differentially expressed genes and metabolites in liver tissue to predict the potential link between cholecystectomy and NAFLD.

In the early phase following cholecystectomy, protein processing in the endoplasmic reticulum and RAS in the liver are possibly activated to promote damaged tissue repair and regeneration (Fig. 1C). In the late post-cholecystectomy period, pyruvate metabolism, citrate cycle, steroid and primary bile acid biosynthesis were inhibited (Fig. 2C). Pyruvate metabolism represents a pivotal pathway in both glycolysis and oxidative phosphorylation. It plays a pivotal regulatory role in maintaining systemic energy homeostasis and material balance^35^. The tricarboxylic acid (TCA) cycle, a significant component of mitochondrial oxidative metabolism is a pivotal nexus of catabolic and anabolic processes. The liver plays a central role in the processes of lipogenesis, gluconeogenesis and cholesterol metabolism^36^. Both pyruvate metabolism and the citric acid cycle are simultaneously inhibited, representing a dual paralysis of the metabolic “engine” and “hub.” This signifies the liver’s loss of its own energy production and synthetic functions. In essence, this results in impaired fat synthesis, disrupted fatty acid oxidation, and reduced production of glutamic acid, aspartic acid, heme, purines, pyrimidines, and other compounds, which has a systemic effect on the body. The majority of cholesterol is embedded within the lipid bilayer, with some members serving as precursors for various signaling pathways. In addition to its role in membrane structure, cholesterol serves as a metabolic precursor for a variety of signaling molecules, including oxysterols, steroids and bile acids^37^. The toxic effects of lipids and bile acids have been identified as contributing factors to NAFLD. In contrast, cholesterol intake has been shown to offer protection against bile acid liver toxicity. The equilibrium between hepatic cholesterol and bile acid levels may possess prognostic value in the context of liver disease progression and trajectory^38^. In this study, the DEGs and DEMs between Cocy30 and Cocy7 were enriched in cholesterol metabolism in the Cocy30 group, indicating that Cholecystectomy is a contributing factor to altered hepatic cholesterol homeostasis.

Primary bile acid biosynthesis in liver constitutes the predominant route of cholesterol catabolism, accounting for approximately 50% of the daily total^39^. The bile acid biosynthetic pathway comprises two primary pathways: the classical pathway and the alternative pathway. Cholesterol 7α-hydroxylase (CYP7A1) is a pivotal enzyme that catalyses the initial rate-limiting step in the classical bile acid biosynthesis pathway^40^. The level of CYP7A1 mRNA in the Cocy30 group increased significantly. This may be due to compensatory transcriptional upregulation resulting from impaired bile acid excretion^41^. It has been demonstrated that, in enterocytes, bile acids bind to FXR, thereby inducing the production and secretion of fibroblast growth factor 19 (FGF19). In turn, FGF19 regulates hepatic bile acid synthesis^42,43^. FGF19 has been shown to bind to a complex of fibroblast growth factor receptor 4 (FGFR4) and beta-Klotho on hepatocytes, thereby inhibiting the bile acid synthesis enzyme CYP7A1 and regulating bile acid production via a negative feedback mechanism^43^. Following cholecystectomy, bile flows continuously and in a diluted state into the duodenum. Bile acids returning to the liver maintain persistently low concentrations, potentially creating the illusion of bile acid deficiency. This activates the FXR signaling pathway in hepatocytes, thereby promoting the transcription of CYP7A1. However, due to the inhibition of energy metabolism processes, such as pyruvate oxidation, the sustained bile secretion and synthesis processes require energy (ATP) and substrates (cholesterol, NADPH). This continuous demand may alter the metabolic state of hepatocytes, resulting in limited synthesis efficiency, despite the CYP7A1 gene being “turned on.”

ATP-binding cassette (ABC) transporter pathway is enriched by DEMS in the Cocy30 group (Supplementary Table D-2). ABC transporters bind to ATP and use its energy to transport various molecules, such as sugars, amino acids and proteins, as well as metal ions, across cell membranes^44^. ABC transporters in the liver play important roles in maintaining bile flow and bile acid circulation and preventing hepatocytes from becoming toxic due to bile acid overaccumulation^45^. Bile acids, particularly the hydrophobic ones, are a major source of chronic inflammatory stress in liver diseases, which could eventually lead to primary liver cancer^45^. Following cholecystectomy, there is an inhibition of primary bile acid synthesis (Fig. 5C). Concurrently, however, there is a considerable increase in bile acid levels (Supplementary Table D-1), possibly due to abnormal ABC transporters.

The present findings suggested that Cholecystectomy leads to an imbalance in hepatic metabolic homeostasis, characterized by inflammatory responses, mitochondrial dysfunction and disordered cholesterol metabolism.

The current study has been limited by the use of UPLC-MS for the detection of liver samples, which has led to the almost undetectable presence of certain low-nonpolar metabolites. In addition, male mice were solely included in the current study, and male and female data were not compared. the effect of both sexes should be conducted as future work.

## Conclusion

In this study, an integrative analysis of the transcriptome and metabolome of the mouse liver tissue post-cholecystectomy was used to assess differences between early and late stages of cholecystectomy. Following cholecystectomy, the liver may undergo a series of subclinical pathological changes, including inflammation, protein processing in the endoplasmic reticulum, suppressed mitochondrial energy metabolism, and dysregulated cholesterol metabolism. Our findings provided a deeper understanding of cholecystectomy and risk of NAFLD.

## Supplementary Information

Below is the link to the electronic supplementary material.

Supplementary Material 1

Supplementary Material 2

Supplementary Material 3

Supplementary Material 4

Supplementary Material 5

Supplementary Material 6

Supplementary Material 7

## Data Availability

The data presented in this study are available on request from the corresponding author due to privacy and ethical considerations.
