# Supplementary figures and images for: Gallbladder removal induces hepatic transcriptional and metabolic shifts with cholesterol dysregulation as a key feature
Source: Sci Rep. 2026 Apr 6;16:16087. doi: 10.1038/s41598-026-46659-8 (PMC13199440; doi:10.1038/s41598-026-46659-8)

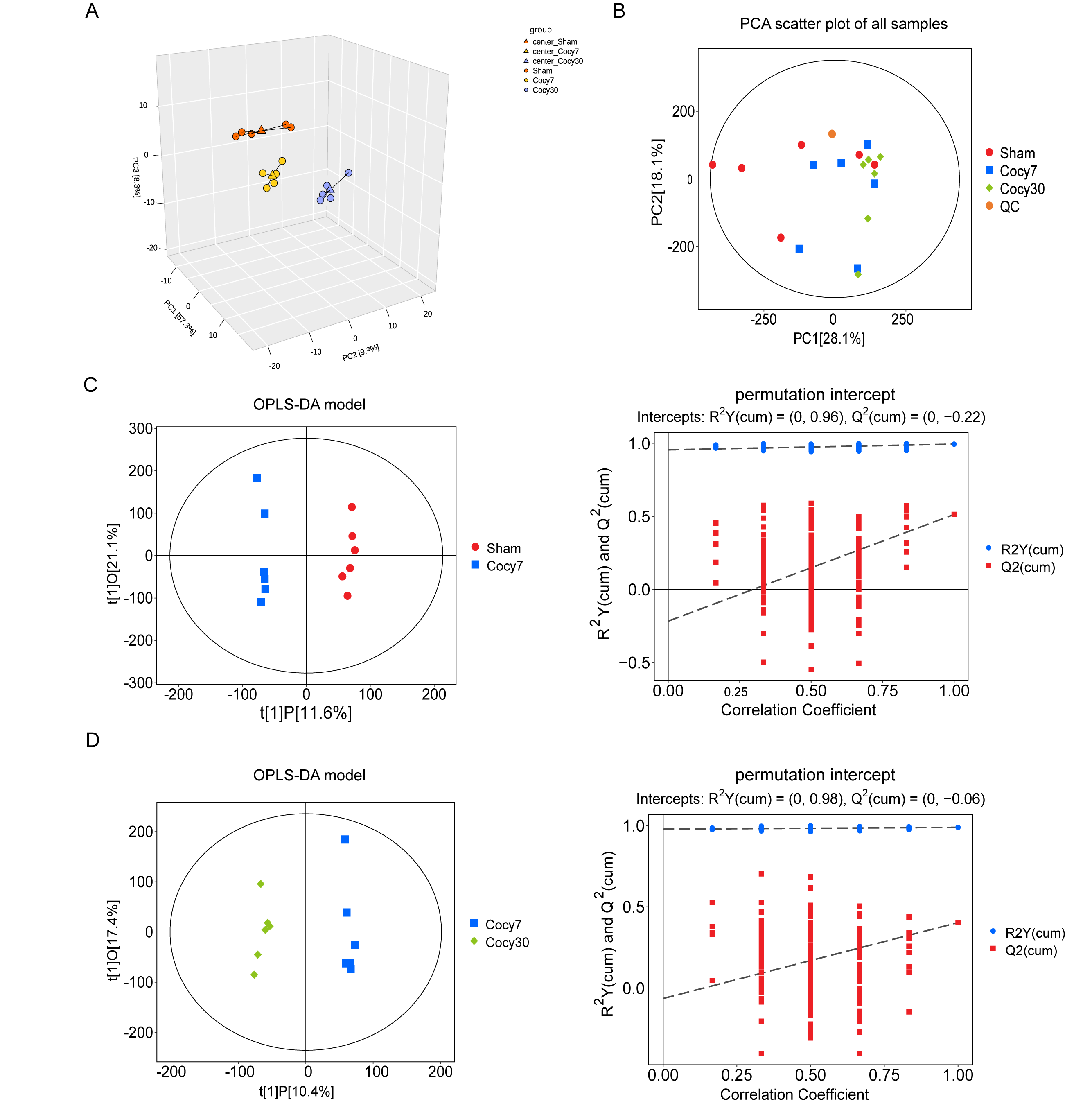

Supplement: Supplementary file 1 — Supplementary Material 1 [file 41598_2026_46659_MOESM1_ESM.tif]
