# Supplementary material for: Gallbladder removal induces hepatic transcriptional and metabolic shifts with cholesterol dysregulation as a key feature
Source: Sci Rep. 2026 Apr 6;16:16087. doi: 10.1038/s41598-026-46659-8 (PMC13199440; doi:10.1038/s41598-026-46659-8)
Supplement: Supplementary file 2 — Supplementary Material 2 [file 41598_2026_46659_MOESM2_ESM.docx]

**Supplementary Figure 1. Quality control data of RNA-seq and metabolomics analysis.** (A)3D Principal Components Analysis (PCA) plot of all samples from the transcriptomics analysis: The x-axis represents the first principal component, the y-axis represents the second principal component, and the z-axis represents the third principal component; (B)PCA plot of all samples from the metabolomic analysis; (C)The scatter plot (left panel) and permutation plot tests (right panel) of the OPLS-DA model for the Sham versus Cocy7 group; (D)The scatter plot (left panel) and permutation plot tests (right panel) of the OPLS-DA model for the Cocy7 versus Cocy30 group.
